# The History of the Absorbent System, Part the First, Containing the Chylography or Description of the Human Lacteal Vessels, with the Different Methods of Discovering, Injecting, and Preparing Them, and the Instruments Used for These Purposes; Illustrated by Figures

**Published:** 1784

**Authors:** 


VI. *The History of the absorbent System, Part the first, containing the Chylography or description of the human lacteal vessels, with the different methods of discovering, injecting, and preparing them, and the instruments used for these purposes; illustrated by figures. By* John Sheldon, *Surgeon, Professor of anatomy in the royal academy of Arts; and Lecturer of anatomy and surgery.* 4to. Cadell, *London,* 1784. 52 pages, with six copper plates, 1 l. 1 s. in boards.

IN the late Mr. Hewson's work on the Lymphatics we have no representation of the lacteals, nor of the lymphatics of the different viscera of the thorax or abdomen; the lymphatic vessels of the viscera, and the lacteals in the human subject, having been but very imperfectly known even at the late period at which Mr. Hewson's treatise was published. The work now before us is intended to supply this deficiency. The author proposes to correct the errors of former writers on this subject, and to give figures from nature of those parts of the lymphatic system in the human body, which have been either erroneously described or recently discovered.

In

In this firſt part of his work, after giving a conciſe hiſtory of the lymphatic ſyſtem, he deſcribes the method of diſcovering, injecting, diſſecting, and preparing the abſorbent veſſels, and then treats of the diſcovery of the lacteals, the ſtructure of their coats, the *ampullulæ Lieberkuhnii*, and laſtly, the manner in which nature produces abſorption in the lymphatic ſyſtem. Theſe deſcriptions are illuſtrated by five very elegant engravings; the ſixth plate exhibits the inſtruments neceſſary for injecting the lymphatics.

In a future part of the work, (moſt of the plates for which are ſaid to be already engraved) Mr. Sheldon means to deſcribe the lymphatics of each particular viſcus, where he has been able to diſcover them by injection or otherwiſe. He promiſes alſo to give a view of the lymphatics of the upper and lower extremities in their natural ſize; and as a ſupplement to the work, to add ſpecimens of the lacteal veſſels in quadrupeds, birds, amphibious animals, and fiſhes.

